# ONC201-Induced Mitochondrial Dysfunction, Senescence-like Phenotype, and Sensitization of Cultured BT474 Human Breast Cancer Cells to TRAIL

**DOI:** 10.3390/ijms232415551

**Published:** 2022-12-08

**Authors:** Artem Mishukov, Irina Odinokova, Ekaterina Mndlyan, Margarita Kobyakova, Serazhutdin Abdullaev, Vitaly Zhalimov, Xenia Glukhova, Vasiliy Galat, Yekaterina Galat, Anatoly Senotov, Roman Fadeev, Artem Artykov, Marine E. Gasparian, Marina Solovieva, Igor Beletsky, Ekhson Holmuhamedov

**Affiliations:** 1 Institute of Theoretical & Experimental Biophysics, Russian Academy of Sciences, Pushchino 142290, Russia; 2 Center for Theoretical Problems of Physicochemical Pharmacology RAS, Moscow 119991, Russia; 3 Institute of Cell Biophysics, Russian Academy of Sciences, Pushchino 142290, Russia; 4 ARTEC Biotech Inc., Chicago, IL 60047, USA; 5 Shemyakin-Ovchinnikov Institute of Bioorganic Chemistry RAS, Moscow 117997, Russia

**Keywords:** ONC201, BT474, mtDNA, changes in the surface proteins expression, senescence, NK-mediated killing of BT474 cells

## Abstract

ONC201, the anticancer drug, targets and activates mitochondrial ATP-dependent caseinolytic peptidase P (ClpP), a serine protease located in the mitochondrial matrix. Given the promise of ONC201 in cancer treatment, we evaluated its effects on the breast ductal carcinoma cell line (BT474). We showed that the transient single-dose treatment of BT474 cells by 10 µM ONC201 for a period of less than 48 h induced a reversible growth arrest and a transient activation of an integrated stress response indicated by an increased expression of CHOP, ATF4, and GDF-15, and a reduced number of mtDNA nucleoids. A prolonged exposure to the drug (>48 h), however, initiated an irreversible loss of mtDNA, persistent activation of integrated stress response proteins, as well as cell cycle arrest, inhibition of proliferation, and suppression of the intrinsic apoptosis pathway. Since Natural Killer (NK) cells are quickly gaining momentum in cellular anti-cancer therapies, we evaluated the effect of ONC201 on the activity of the peripheral blood derived NK cells. We showed that following the ONC 201 exposure BT474 cells demonstrated enhanced sensitivity toward human NK cells that mediated killing. Together our data revealed that the effects of a single dose of ONC201 are dependent on the duration of exposure, specifically, while short-term exposure led to reversible changes; long-term exposure resulted in irreversible transformation of cells associated with the senescent phenotype. Our data further demonstrated that when used in combination with NK cells, ONC201 created a synergistic anti-cancer effect, thus suggesting its possible benefit in NK-cell based cellular immunotherapies for cancer treatment.

## 1. Introduction

ONC201 is an experimental anticancer drug from the imipridone class of compounds [1,2], currently in multiple clinical trials in the United States <https://clinicaltrials.gov/ct2>. To date, the only direct target identified for ONC201 is the mitochondrial caseinolytic protease P (ClpP) [3,4,5], and effects on mitochondrial metabolism have been described [3,5]. However, despite these findings, the exact mechanism of ONC201 action is not well-understood [5,6,7,8,9]. In addition to ONC201’s cytotoxic effects on several cancer cell lines [3,5,6,10,11], there are indications that the properties of ONC201 may be due to its cytostatic effects [6,10]. Recently, it was shown that ONC201 causes significant morphological changes in the structure of mitochondria, disruption of mitochondrial morphology with a shift towards fragmentation, inhibition of mitochondrial respiration, and loss of mtDNA [3,6,7,12]. Many pharmacological drugs (especially antiviral and chemotherapeutic drugs) are capable of inducing mitochondrial dysfunction through a decrease in cellular mtDNA and associated loss of 13 essential proteins of oxidative phosphorylation [13,14,15,16], which are encoded by mtDNA and are synthesized in the mitochondrial matrix [16,17,18].

Disruption of the synthesis of mtDNA encoded proteins and dysfunction of mitochondrial oxidative phosphorylation are associated with a variety of pathologies [19,20]. Surprisingly, such dramatic changes in the cellular proteostasis and energetics are not associated with noticeable damage and cell survival [12]; on the contrary, this drug inhibits proliferation, cell cycle progression and causes a cytostatic effect.

We demonstrate in the current work that ONC201, an agent which possesses a prominent antiproliferative effect on cultured cells and causes cell cycle arrest, does not show any signs of cytotoxicity even at relatively high doses, as compared with IC50. Available data demonstrate that the reported effects of ONC201 in cancer cells fall into two groups of effects: (a) cultured cells in which exposure to ONC201 results in induction of apoptotic cell death [3,5,8,9,19], and (b) the non-apoptotic effects of ONC201 resulting in the arrest of proliferation and the cell cycle, and absence of the cytotoxicity [4,8,9,10]. The absence of the cytotoxicity and cell killing by ONC201 in BT474 cells (as well as other cell types) raises the questions on reversible and irreversible consequences of ONC201 treatments. Herein, we focused on the dose- and time-dependent effects of ONC201 on cells’ proliferation, cell cycle progression and expression of stress-associated proteins, and the sensitivity of ONC201-treated BT474 cells toward NK-mediated cell killing, in connection with the duration of the exposure of BT474 cells to the drug.

The aim of the current study was to analyze the dependence of ONC201 effects on the duration of single-dose drug treatment of BT474 cells. Herein, we examined the short-term and long-term consequences of a single-dose exposure of cultured BT474 cells to ONC201. Specifically, using different protocols/regiments of treatment of BT474 cells with ONC201, we evaluated the ability of this drug to activate apoptotic and immunogenic cell death pathways, which is of key importance in the development of the effective strategy for cancer therapy. Surprisingly, the duration of the exposure of BT474 cells to a given, non-toxic dose of ONC201 was a very critical parameter of treatment. Short-time exposure (24 h and less) of cells to ONC201 and subsequent washout of the drug for 120 h in drug-free media were associated with almost complete reversibility of all observed changes in the rate of proliferation, cell cycle progression, and changes in the level of expression of characteristic stress-induced proteins. On the contrary, prolonged exposure of these cells to the same concentration of ONC201 (72 h and longer) and subsequent incubation of cells in drug-free media (washout of the drug) resulted in irreversible cell cycle arrest, complete inhibition of proliferation, and irreversible changes in the level of expression of stress proteins. Moreover, these irreversible changes were accompanied by enhanced susceptibility of ONC201-treated cells to killing by human NK-cells. In conclusion, our data demonstrate that the long-lasting consequences of ONC201 treatment result in transformation of BT474 cells resulting in forcing them into a senescent-like phenotype with the loss of the ability to proliferate.

## 2. Results

### 2.1. Anti-Proliferative Effect of ONC201 in Cultured Human Breast Cancer BT474 Cells

**Figure 1:**
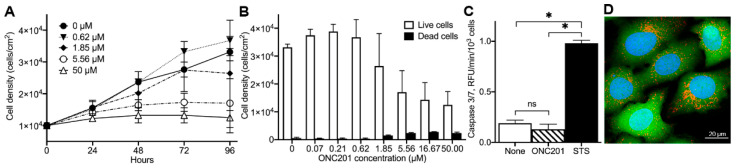
Effect of ONC201 on growth, viability, activation of Caspase-3/7, and morphology of BT474 cells. (**A**), Time- and dose-dependent inhibition of cell proliferation by ONC201. (**B**), Dose-dependent suppression of cell growth by ONC201 (96 h exposure). (**C**), Caspase-3/7 activation in untreated cells (None), cells treated for 72 h with 10 µM ONC201 (ONC201) or 6 h with 0.5 µM staurosporine (STS). Shown are mean +/− SD from at least three independent experiments. Initial plating density of cells at time “zero” was 30,000 cells/cm^2^, * *p* < 0.05, “ns”, non-significant. (**D**), Confocal images of cells demonstrating viability of cells treated with 10 µM of ONC201 for 72 h. Cells were loaded with 2 µg/mL Hoechst 33342 (Blue, cell nuclei), 1 µM Calcein-AM (Green, cytoplasm), and 150 nM Mito Tracker Deep Red (Red, mitochondrial mass) in HBSS buffer for 30 min at 37 °C.

**Figure 2:**
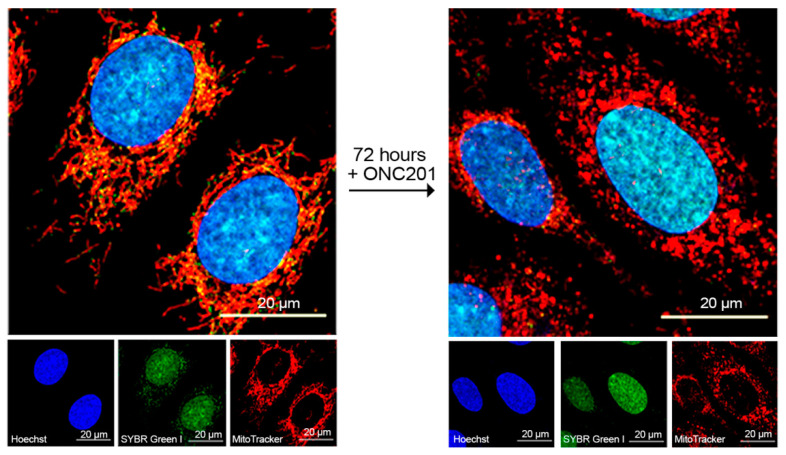
Effect of ONC201 on mitochondrial morphology and mtDNA content of BT474 cells. Confocal fluorescent images of BT474 before (left panel) and after exposure to single-dose 10 µM ONC201 for 72 h (right panel). Cells were loaded with Hoechst 33342 (2 µg/mL), SYBR Green I (1:200,000 dilution), and MitoTracker Deep Red (150 nM) in HBSS buffer for 30 min at 37 °C. The small panels show individual images of nuclei (blue, Hoechst 33342), mitochondrial nucleoids and nuclear DNA (green, SYBR Green 1), and mitochondria (red, Mito Tracker Deep Red).

To examine the effects of ONC201 on breast cancer cell proliferation, viability, and death, we exposed cultured BT474 cells to different concentrations of ONC201 (0–50 µM) for the indicated time (Figure 1A). The total cell number and the number of dead cells were determined as described in Materials and Methods. ONC201 at concentrations of 0–50 µM dose-dependently suppressed the proliferation of these cells without inducing significant cell death (Figure 1B, compare open vs. filled bars). The estimated IC_50_ value for ONC201 in cultured BT474 cells was determined to be around 2 µM, which agrees with IC_50_ determined in previous studies using different cell lines [2,3,4,5]. Contrary to some other cancer cells [5,20,21,22], ONC201 at these concentrations did not activate caspase 3/7 in BT474 cells as demonstrated using a fluorogenic Ac-DEVD-AMC substrate (Figure 2). Caspase 3/7 activity in untreated cells (None) was determined to be 0.19 ± 0.03 RFU/min/10^3^ cells, and in cells treated with 10 µM ONC201 for 72 h (ONC201) was 0.13 ± 0.05 RFU/min/10^3^ cells (Figure 1C, open and stripped bar, respectively, n = 3, *p* = NS). By contrast, staurosporine, an established apoptosis inducer, demonstrated substantial activation of caspase-3/7 in cultured BT474 cells and reached 0.98 ± 0.03 RFU/min/10^3^ cells (Figure 1C, filled bar, n = 3, *p* < 0.05). Consistent with the absence of apoptotic cell death, exposure of BT474 to ONC201 did not affect nuclear morphology or cell membrane integrity, as shown using Hoechst 33342, and retention of Calcein fluorescence (Figure 1D). Thus, ONC201 demonstrates dose-dependent anti-proliferative activity in BT474, without noticeable cytotoxicity and/or activation of caspase 3/7.

### 2.2. Long-Lasting Effect of ONC201 on the Number of Mitochondrial Nucleoids and Mitochondrial Morphology

Next, we examined the effects of ONC201 on the number of mitochondrial nucleoids and mitochondrial morphology. Figure 2 shows confocal images of BT474 cells treated with 10 µM ONC201 and loaded with Hoechst 33342 to visualize nuclei (blue color), SYBR Green-1 (green color) to visualize mitochondrial nucleoids, and Mito Tracker Deep Red (red color) to mark mitochondrial mass. These images demonstrate a decline in the numbers of mitochondrial nucleoids and changes in the mitochondrial morphology in BT474 cells exposed to 10 µM ONC201 (Figure 2 compares images on left panel and right panel). In untreated cells, mitochondrial nucleoids co-localized together with tubular and highly branched mitochondrial networks and demonstrated the intramitochondrial localization of nucleoids (Figure 2, left panel, red and green fluorescing structures). Treatment of cells with 10 µM ONC201 for 72 h induced mitochondrial fragmentation of initially thread-like mitochondria (red fluorescence) and decreased the number of mitochondrial nucleoids (Figure 2, right panel, green punctate fluorescing structures in the cytosol), which indicate mitochondrial damage.

Since ONC201 treatment induces mitochondrial damage and inhibits proliferation without induction of cell death, we examined the long-lasting consequences of a single-dose treatment on the number of mitochondrial nucleoids and mitochondrial morphology. For this purpose, we used the treatment protocol described in Materials and Methods and illustrated in Supplementary Figure S1. Briefly, cells were treated with a 10 µM single dose of ONC201 for 24, 48, and 72 h; then, the drug was removed from the incubation media, and cells were incubated in the drug-free media for 120 h (5 days) with regular (every 24 h) incubation media replacement.

**Figure 3:**
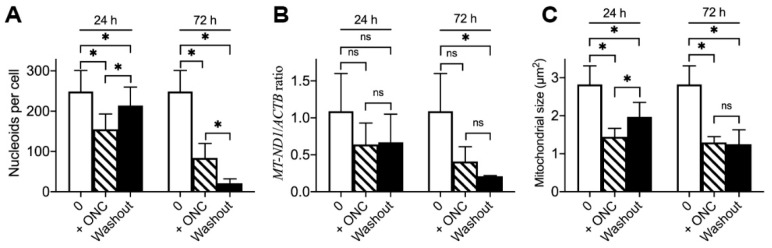
Long-lasting consequences of a single-dose treatment with ONC201 on the mitochondrial nucleoids, mtDNA, and mitochondrial morphology. (**A**), Average number of mitochondrial nucleoids per cell before, following single-dose 10 µM ONC201 treatment (+ONC) and subsequent washout (washout); (**B**), Ratio of mtDNA gene *MT-ND1* to nDNA encoded gene *ACTB* normalized to control (qPCR data); (**C**), Average size of mitochondria in 10 µM ONC201 treated cells. Shown are the mean +/− SD obtained from at least three independent experiments. Statistics conducted using one-way ANOVA, post-hoc Bonferroni test, * *p* < 0.05, “ns”, non-significant.

Quantification of obtained images (Supplementary Figure S2) demonstrated that long-lasting consequences of ONC201 single-dose treatment depend on the duration of BT474 cell exposure to the drug. The 24 h exposure to ONC201 resulted in a decline in the number of mitochondrial nucleoids from 249 ± 52 to 155 ± 38 nucleoids per cell, and subsequent 120 h washout partially restored the number to 214 ± 46 nucleoids per cell (Figure 3A, 24 h). The 48 h exposure of BT474 cells to a single dose of ONC201 (10 µM) resulted in a decline in the number of mitochondrial nucleoids from 249 ± 52 (in control) to 78 ± 28 nucleoids per cell, and, after a subsequent 120 h washout, the average number of mitochondrial nucleoids was 91 ± 72 nucleoids per cell. On the contrary, 72 h exposure to ONC201 led to a greater decline in the number of mitochondrial nucleoids (from 249 ± 52 to 84 ± 36 nucleoids per cell), and the subsequent washout resulted in a further drop up to 21 ± 11 nucleoids per cell (Figure 3A, 72 h). The ONC201-dependent reduction in nucleoids in BT474 cells (Figure 3A) correlated with the decreased content of mitochondrial DNA (mtDNA) in BT474 cells as demonstrated by q-PCR (Figure 3B). The ratio of mtDNA-encoded gene *MT-ND1* (NADH-ubiquinone oxidoreductase chain 1) normalized to nuclear DNA encoded gene *ACTB* (*β*-actin), demonstrating that 24 h ONC201 treatment decreased this ratio from 1.09 ± 0.51 in non-treated BT474 cells to 0.64 ± 0.29 and remained at the same level after washout (Figure 3B, 24 h), whereas 72 h treatment resulted in a drop to 0.41 ± 0.20 with a further decrease to 0.21 ± 0.01 after washout (Figure 3B, 72 h). Along with changes in the content of mitochondrial nucleoids, ONC201 treatment induced fragmentation of the mitochondrial network. A 24 h exposure to the drug resulted in a decrease in the average size of mitochondria (from 2.82 ± 0.49 µm^2^ to 1.45 ± 0.22 µm^2^), and a subsequent washout led to an increase in the average mitochondrial size to 1.97 ± 0.38 µm^2^ (Figure 3C, 24 h) when 72 h exposure demonstrated a similar drop in the mitochondrial size (to 1.29 ± 0.15 µm^2^), which remained at the same level after washout (Figure 3C, 72 h). Treatment of BT474 cells with ONC201 for 192 h (8 days) resulted in similar results to 72 h exposure + 120 h washout. These results demonstrate that long-term (72 h) exposure of BT474 cells to a single-dose ONC201 induces irreversible mitochondrial damage: sustained mitochondrial fragmentation and depletion of mitochondrial nucleoids and mtDNA.

### 2.3. Sustained Inhibition of Proliferation, Activation of the Integrated Stress Response (ISR), and Depletion of Mitochondrial Proteins in Cultured BT474 Cells Exposed to a Single Dose of ONC201

**Figure 4:**
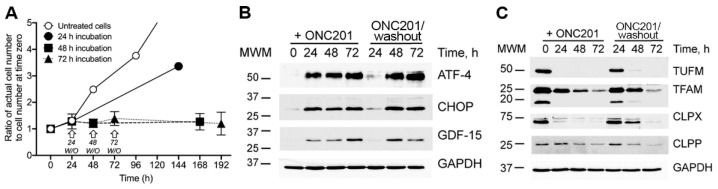
Time-dependent effect of single dose ONC201 exposure of BT474 cells on proliferation and expression of markers of ISR. (**A**), Proliferation of BT474 exposed to 10 µM ONC201 and following washout (marked with arrows). (**B**), Representative Western blots of markers of ISR (ATF4 and CHOP) and stress-protein GDF-15 following treatment with 10 µM of ONC201 (+ONC201) and subsequent 120 h washout (−ONC201(washout)); (**C**), Representative Western blots of mitochondrial proteins following treatment with 10 µM of ONC201 (+ONC201) and subsequent 120 h washout (−ONC201(washout)). Shown are mean ± SD (**A**) and representative Western blots (**B**,**C**) from at least three independent experiments.

We observed that ONC201 inhibited BT474 cell proliferation (Figure 1A,B) and had long-lasting effects on the mitochondria, which were dependent on the duration of cell exposure to drug (Figure 3). Along with effects on the mitochondrial characteristics, ONC201 treatment differentially affected proliferation of BT474 cells. Untreated cells proliferated with an estimated doubling time of 32–36 h (Figure 4A, open circle symbols), the doubling time of cells exposed to 10 µM ONC201 for 24 h, and subsequently incubated in drug-free incubation media for 120 h, increased to ~79–85 h (Figure 4A, filled circle symbols). Increasing the initial exposure of cells to ONC201 (48 h and longer), resulted in the near complete suppression of cell proliferation even after removal of drug and incubation of cells in drug-free media (Figure 4, filled square and triangle symbols).

Previous studies showed that ONC201 arrested cell growth and increased the ISR in breast cancer cells [4]. This long-term response to treatment with ONC201 was observed at the level of the induced integrated stress response (ISR) as determined by increases in multiple ISR-associated proteins [23,24]. Treatment of BT474 cells with a single dose of ONC201 resulted in enhanced expression of ATF4, CHOP, and GDF-15 during exposure to the drug (Figure 4B, lanes under +ONC201), which was reversed after washout of the drug and incubation of cells in drug-free media: 24 h exposure to ONC201 resulted in transient and reversible activation of stress proteins, whereas longer (48 and 72 h) exposure resulted in persistent, irreversible activation of ATF4, CHOP proteins and demonstrated only partial reversibility of GDF-15 expression (Figure 4B, lanes under −ONC201 (washout)).

Treatment of breast cancer cells with ONC201 leads to degradation of mitochondrial nucleoids and mtDNA (Figure 3A,B) as previously demonstrated [3,5,12]. Similarly, the treatment of BT474 cells with ONC201 resulted in the decline in TUFM (mitochondrial elongation factor) and TFAM (mtDNA packaging and transcription factor) as shown in Figure 4C. Exposure of BT474 cells to a single dose of ONC201 (10 µM, 24 h) or longer (48 and 72 h) resulted in the almost complete disappearance of TUFM (Figure 4C, TUFM, +ONC201). However, short-term treatment with ONC201 (24 h), followed by the 120 h washout, resulted in the restoration of this protein (Figure 4C, TUFM, −ONC201/washout). By contrast, extended exposure to ONC201 (48 h and 72 h) resulted in the irreversible decline in the expression of TUFM (Figure 4C, TUFM, −ONC201/washout). Presented western blot data rather demonstrate a more substantial decrease in TUFM expression in ONC201 treated cells than the actual remaining level of TUFM under these conditions. Similar results were observed with TFAM, a high-mobility group DNA-binding and bending protein [25], that determines the stability and transcription of mtDNA [16,26,27,28]. In accord with the time-dependent disappearance of mitochondrial nucleoids from BT474 cells exposed to single dose ONC201 (Figure 2 and Figure 3A), the level of TFAM protein in ONC201-treated cells depended on the duration of the treatment and gradually declined (Figure 4C, TFAM, +ONC201). Loss of TFAM was slower than that observed for TUFM, and coincided with ONC201-induced disappearance of mitochondrial nucleoids and degradation/depletion of mtDNA (Figure 3A,B).

Interestingly, the treatment of BT474 cells with ONC201 also affected the level of ClpP, the immediate target of ONC201 [3,4,5] and its counterpart, the ATP-dependent unfoldase subunit ClpX, which is responsible for recognition and targeting of misfolded or damaged proteins to ClpP for degradation [29]. The pattern of ONC201-induced decreases in ClpP, ClpX, TFAM, and TUFM proteins was opposite of that observed for the stress proteins. Short-term (24 h) exposure of cells to the drug and subsequent washout demonstrated the reversible decline of these proteins, which returned to initial levels following the 120 h washout and incubation of these cells in drug-free media (Figure 4C, +ONC201 vs. −ONC201/washout). On the contrary, the long-term (48 h and 72 h) exposure of these cells to the drug demonstrated irreversible loss of these proteins after 48 and 72 h ONC201 treatment (Figure 4C).

Thus, exposure of BT474 cells in the culture to ONC201 induced ISR regardless of the duration (24, 48, or 72 h), as demonstrated in Results Section 2.1, by induction of stress response proteins ATF4, CHOP, and GDF-15. The increase in the ISR was reversible after short-term exposure (24 h), whereas longer exposure (48 and 72 h) resulted in irreversible elevation of these proteins, which persisted for 120 h after removal of the drug. Taken together, our observations indicate that the long-term exposure (for a duration exceeding the doubling time of BT474 cells) to ONC201 revealed the existence of cellular “memory”, manifested in sustained arrest of proliferation, persistent and elevated expression of stress markers, and the corresponding loss of TUFM and TFAM.

### 2.4. Effect of Single Dose Treatment of Cells with ONC201 on the Cell Cycle Distribution of BT474 Cells

**Figure 5:**
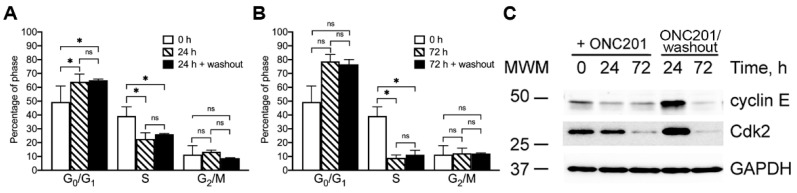
ONC201 induces G_1_/S cell cycle arrest of BT474 cells. (**A**), Distribution of BT474 cells in cell cycle phases before (0 h), after 24 h treatment with 10 µM ONC201, and after subsequent 120 h washout. (**B**), Distribution of BT474 cells in cell cycle phases before (0 h), after 72 h treatment with 10 µM ONC201, and after subsequent 120 h washout. (**C**), Representative Western blots of cell cycle regulatory proteins following treatment with 10 µM ONC201 (+ONC201) and subsequent 120 h washout (ONC201/(washout). Shown are mean ± SD (**A**,**B**) and representative Western blots (**C**) from at least three independent experiments. The data were analyzed using one-way ANOVA with post-hoc Bonferroni test, * *p* < 0.05, “ns”, non-significant.

Treatment of cancer cells with ONC201 inhibits cell proliferation and causes cell cycle arrest [8,9,30]. We combined our investigation of the anti-proliferative activity of ONC201 in BT474 cells with the analysis of ONC201-induced redistribution of cells in the phases of the cell cycle. Short-term (24 h) and long-term (72 h) treatment of BT474 cells with ONC201 inhibited cell cycle progression and caused similar changes in the cell cycle distribution of cells (Figure 5A,B). The initial distribution of BT474 (0 h, open bars) was 49.4 ± 11.5% (G_0_/G_1_), 39.2 ± 6.6% (S), and 11.4 ± 6.4% (G_2_/M), which shifted after treatment of cells for 24 h with single-dose ONC201 (Figure 5A, 24 h) to 64.1 ± 5.6% (G_0_/G_1_), 22.6 ± 4.5% (S), and 13.4 ± 1.2% (G_2_/M). The distribution of cells treated for 24 h with the drug and subsequently incubated in drug-free media for 120 h (Figure 5A, 24 h + washout) did not result in statistical changes in the distribution of the cell cycle phases. 

As compared with the initial distribution of cells (0 h), long-term (72 h) ONC201 treatment demonstrated an increased accumulation in the G_0_/G_1_ phase (78.7 ± 5.1%) with a greater decline in the S phase (9.0 ± 2.1%), compared to 24 h treatment, without noticeable changes in the cell number in the G_2_/M phase (12.3 ± 3.8%). The subsequent washout of the drug from these cells for 120 h did not result in further changes in the cell cycle distribution, which remained at the same level (Figure 5B, 72 h + washout). Thus, analysis of the cell cycle distribution of BT474 cells exposed to a single dose of ONC201 demonstrated a significant accumulation of cells in the G_0_/G_1_ phase, with a concomitant decline in the S phase, indicating that this treatment prevented cells from entering into the S phase. These observations are in line with the decreased amount of the cell cycle dependent kinase 2 (Cdk2) and cyclin E (Figure 5C), proteins regulating the S phase entry [31]. Incubation of BT474 cells with single dose ONC201 for 24 and 72 h resulted in a time-dependent and gradual decline in expression of both CDK2 and cyclin E, which is in line with the observed cell cycle arrest (Figure 5C). Washout of the drug from cells treated with a single dose of ONC201 for 24 h reversed and restored Cdk2 and cyclin E (Figure 5C, −ONC201 (washout), 24 h), which is in line with the restored proliferation activity of these cells. However, 72 h exposure of cells to a single dose of the drug resulted in irreversible loss of these proteins that was not restored after washout of the drug (Figure 5C, −ONC201(washout)). Thus, short-term (24 h) exposure of BT474 cells to ONC201 results in reversible cell cycle arrest and a decline in the expression of Cdk2 and cyclin E, while long-term (72 h) exposure of cells to the drug resulted in irreversible degradation of Cdk2/cyclin E and persistent G_1_/S cell cycle arrest.

### 2.5. ONC201 Treatment Time-Dependently Suppresses Mitochondrial Respiration via Complex I and Complex II

It was reported that ONC201 treatment results in a decline in the level of subunits of respiratory chain complexes [3,32]. ONC201-induced degradation of respiratory chain subunits could be responsible for the decreased cellular respiration observed after ONC201 exposure [3,5,33] in a variety of cell lines. Based on the observation that treatment with ONC201 selectively depletes mitochondrial nucleoids and mtDNA [3,5,12], we expected to observe impaired activity of mitochondrial respiratory chain complexes and alteration of mitochondrial oxidative phosphorylation in BT474 cells [17,18,34,35]. We studied the effects of ONC201 on the activity of mitochondrial respiratory chain Complex I and Complex II using a digitonin-mediated permeabilization of the plasma membrane to gain direct access to intact intracellular mitochondria [36]. We compared the rate of two major parameters of mitochondrial oxidative phosphorylation: State 3 respiration (ADP-stimulated) and the maximal activity of the respiratory chain (uncoupled, 2,4-DNP stimulated) rate of oxygen consumption in BT474 cells treated with ONC201.

**Figure 6:**
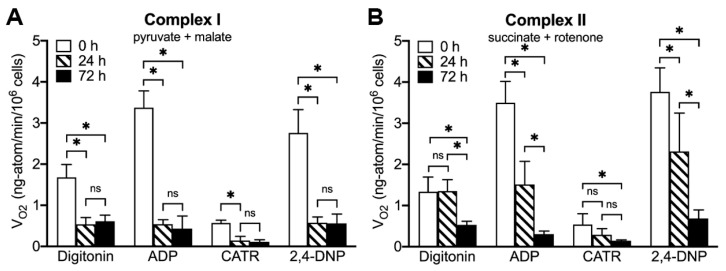
ONC201-induced suppression of mitochondrial respiratory chain activity. (**A**), Rate of oxygen consumption of BT474 cells, oxidizing Complex I substrates. (**B**), Rate of oxygen consumption of BT474 cells, oxidizing Complex II substrates. Open bars demonstrate respiration of untreated cells (0 h exposure to drug); Striped bars reflect the respiration of short-term treated cells (24 h); and Filled bars show the effect of ONC201 on the activity of respiratory complexes after 72 h of exposure. Oxygen consumption rate by intracellular mitochondria was assessed following permeabilization of plasma membrane with 0.005% digitonin and subsequent additions of 200 µM ADP, 1 µM Carboxyatractyloside (CATR) and 25 µM 2,4-DNP. Shown are mean ± SD from at least three independent experiments. Statistical significance was estimated using one-way ANOVA with post-hoc Bonferroni test, * *p* < 0.05, “ns”, non-significant.

Unlike the known inhibitors of mitochondrial oxidative phosphorylation (such as Rotenone, Antimycin A, Oligomycin, KCN), the inhibitory effect of ONC201 required the incubation of living cells, and the drug had no immediate effect on the respiration of isolated mitochondria (Supplementary Figure S3). The inhibitory effect of ONC201 on respiration gradually developed during incubation of cells with the drug, confirming that ONC201-induced degradation of respiratory complexes could be responsible for respiratory dysfunction [3,32,33]. The inhibitory effect of ONC201 in BT474 cells was crucially dependent on the duration of the drug exposure and caused quicker changes in the activity of Complex I as compared with the activity of Complex II (Figure 6).

The Complex I basal activity in digitonin-permeabilized cells, treated with ONC201 for 24 h, was suppressed by ~70% and decreased from the initial 1.68 ± 0.31 to 0.54 ± 0.16 ng-atoms O/min/10^6^ cells in ONC201-treated cells, without further changes after 72 h of treatment (Figure 6A, digitonin). Similarly, ADP-stimulated and uncoupled respiration of Complex I decreased after 24 h exposure to ONC201 from 3.37 ± 0.41 to 0.54 ± 0.11 ng-atoms O/min/10^6^ cells and 2.76 ± 0.57 to 0.58 ± 0.14 ng-atoms O/min/10^6^ cells, respectively, without further changes after 72 h of treatment (Figure 6A, ADP and 2,4-DNP). The Complex II basal activity in digitonin-permeabilized cells was not affected after 24 h of exposure but was reduced by ~60% after 72 h of treatment and decreased from the initial 1.34 ± 0.36 to 0.53 ± 0.10 ng-atoms O/min/10^6^ cells (Figure 6B, digitonin). ADP-stimulated and uncoupled respiration of Complex II, in contrast to Complex I, decreased gradually: 24 h exposure to ONC201 resulted in ~50% loss of activity from the initial 3.50 ± 0.52 to 1.51 ± 0.56 ng-atoms O/min/10^6^ cells and from 3.76 ± 0.58 to 2.31 ± 0.93 ng-atoms O/min/10^6^ cells, respectively (Figure 6B, 24 h, ADP and 2,4-DNP). Long-term exposure of BT474 cells to ONC201 resulted in a further decrease in State 3 and uncoupled respiration of Complex II: to 0.31 ± 0.07 ng-atoms O/min/10^6^ cells and to 0.69 ± 0.21 ng-atoms O/min/10^6^ cells, respectively (Figure 6B, 72 h, ADP and 2,4-DNP). Suppression of Complex I and Complex II activity of the mitochondrial respiratory chain in BT474 cells was dependent on the duration of treatment with ONC201, and inhibition of respiratory chain activity of mitochondria persisted following 120 h post-treatment incubation of these cells in drug-free media.

### 2.6. ONC201 Long-Term Treatment Sensitizes BT474 Cells to Cytotoxic Effects of TRAIL and Human NK-Cells

It is well known that long exposure (from hours to days) of cancer cells to the small molecules results in an activation of ISR, induction of a senescence-like state, inhibition of proliferation and cell cycle arrest, elevated expression of stress factors as well as an induction of immunogenic cell death pathways [37,38,39,40]. We extended our work to include and describe long-lasting consequences of ONC201 treatment in BT474 cells, which will complement an induction of persistent ISR (Figure 4B), degradation of mitochondrial nucleoids and mtDNA (Figure 3A,B), suppression of the activity of mitochondrial respiratory chain complexes, and G_1_/S cell cycle arrest (Figure 5B and Figure 6). Long-term (72 h) treatment of BT474 cells with ONC201 resulted in irreversible G_1_/S cell cycle arrest, activation of ISR, and mitochondrial dysfunction, without cytotoxic consequences to cells.

**Figure 7:**
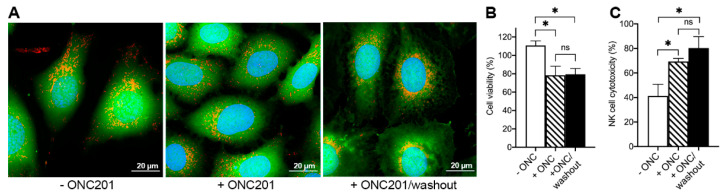
Long-term ONC201 treatment increases the susceptibility of BT474 cells to TRAIL (DR5b) and NK-cells induced killing. (**A**), Confocal images of BT474 before treatment with ONC201 (−ONC201), after 72 h with 10 µM ONC201 (+ONC201), and after subsequent 5-day washout (+ONC201/washout). Cells were loaded with 2 µg/mL Hoechst 33342 (blue, nuclei), 1 µM Calcein-AM (green, cytoplasm), and 150 nM MitoTracker Deep Red (red, mitochondria); (**B**), Viability of BT474 cells pre-treated with ONC201 as in panel (**A**) and subsequently treated with 1000 ng/mL TRAIL for 24 h; C, NK cell cytotoxicity toward BT474 (pretreated with ONC201 as in panel (**A**)). * *p* < 0.05, “ns”, non-significant.

Independent of the duration of treatment with ONC201, BT474 cells retained plasma membrane integrity, as shown by the retention of Calcein dye within cells treated with the drug for 72 h and a subsequent 120 h washout (Figure 7A, green fluorescence). Despite sustained mitochondrial transformations as manifested in the fragmentation of the mitochondrial network and loss of the mtDNA and OxPhos impairment, the cells maintained mitochondrial mass as shown by the mitochondria-specific dye (Figure 7A, red fluorescence).

Intactness of the plasma membrane in ONC201-treated cells, together with previous observations that ONC201 pretreated breast cancer cells are more susceptible to TRAIL and NK-cell mediated death [10], led us to further testing of the sensitivity of ONC210-treated cell toward TRAIL, a well-known inducer of inducers’ apoptotic cell death of cancer cells. Exposure of BT474 cells to 10 µM ONC201 transformed initially TRAIL-insensitive BT474 cells into TRAIL-sensitive cells (Figure 7B). Incubation of BT474 cells in the continuous presence of ONC201 for 72 h and the subsequent washout of the drug for 120 h transformed and sensitized cells to the cytotoxic effects of 1000 ng/mL of TRAIL (Figure 7B, +ONC/washout).

Similarly, BT474 cells pretreated with 10 µM ONC201 for 72 h demonstrated enhanced sensitivity toward human NK-cells mediated killing and an increased cytotoxicity as compared with naïve, untreated cells (Figure 7C). NK-cells’ assay demonstrated increased cytotoxicity of NK cells toward BT474 cells from 41.3 ± 9.5% in untreated BT474 cells (Figure 7C, −ONC201, open bar) to 69.3 ± 2.5% for ONC201-treated cells (Figure 7C, +ONC201, hatched bar). The differences in sensitivity of untreated and ONC21-treated BT474 to NK-cells’ cytotoxicity were retained by ONC201-treated cells even after 120 h washout of the drug when treated cells were maintained in culture for 120 h with a 24 h drug-free media change (Figure 7C, +ONC201/washout, solid bar). 

## 3. Discussion

The recently discovered small molecule ONC201 demonstrating exceptional safety and antitumor activity against multiple cancers is currently under investigation in a number of clinical trials in the United States. The mechanism of action of ONC201 is currently unknown, and although some data demonstrate that ONC201 causes cytotoxic effects and the mechanism of its action based on TRAIL-mediated apoptotic cell death [2,3,5,6,30,41], there are contrasting data demonstrating that ONC201 is rather cytostatic than cytotoxic and does not induce cell death in some cancer models, particularly in breast cancer cell lines [4,8,33]. Recent studies identified the mitochondrial caseinolytic protease P (ClpP) as the main target for ONC201 and other related analogs [3,4] and suggested that growth inhibition resulted from the activation of ClpP and increased turnover of mitochondrial proteins [33,42]. ONC201-mediated activation of ClpP and depletion of selective mitochondrial proteins were shown to result in decreased activity of respiratory chain complexes I, II, and IV [3,43] and impairment of oxidative phosphorylation [33]. Proteomic analysis of cells treated with imipridones demonstrated massive depletion of intracellular proteins including proteins of the mitochondrial matrix [3,32,42], a reduction in nuclear-encoded TFAM and TUFM, structural and functional regulators of mtDNA transcription [4], indicating the destructive effect of ClpP activation on mitochondrial processes.

In line with observations from other laboratories, we demonstrated using the hormone-receptor positive BT474 cell line [3,5,42,44] that single-dose exposure to ONC201 induced a sustained decrease in the number of mitochondrial nucleoids and degradation of mtDNA, long-lasting activation of stress signaling pathways, and arrest of proliferation [12,45]. We extended these observations to include long-lasting and persistent post-treatment changes observed in single-dose ONC201-treated cells. We demonstrated that a 72 h drug exposure and subsequent incubation of these cells in drug-free media for an additional 120 h with a 24 h media change results in persistent and *irreversible* inhibition of proliferation, in contrast to almost complete restoration of the rate of proliferation of BT474 treated for 24 h (Figure 5A). Interestingly, these changes were reversible in cells exposed to ONC201 for 24 h, and washout of cells from the drug restored the number of nucleoids almost to the initial level (Figure 4A). However, in cells treated with ONC201 for 72 h and longer, subsequent incubation of treated cells in drug-free media did not follow with restoration of the number of mitochondrial nucleoids and mtDNA (Figure 4A,B). A single exposure of BT474 cells to 10 µM ONC201 decreased the total mitochondrial mass as determined from MTDR fluorescence independently on the duration of exposure (Figure 4C).

As expected, single-dose drug exposure of BT474 cells to the drug induced ISR in these cells and elevation of the expression of ATF4, CHOP, and GDF-15, characteristic stress proteins [23,24], which was dependent on the duration of ONC201 exposure (Figure 5B). In 24 h treated cells subsequently subjected to a 120 h washout of drug, the level of expression of stress markers was completely reversible and returned back to the initial (Figure 5B, 24 h comparing +ONC201 with −ONC201). On the contrary, longer treatment of cells (for 48 h and 72 h) with subsequent 120 h washout of the drug using drug-free media resulted in an irreversible increase in the level of stress proteins in these cells (Figure 5B, 48 h and 72 h, comparing +ONC201 with −ONC201). Expression of stress proteins following a single exposure and subsequent 120 h washout persisted only in cells treated with the drug for 48 h and longer (Figure 5C), indicating that even single-dose transient exposure of BT474 cells to ONC201 induces long-lasting and persistent stress.

We also demonstrated that a single dose of ONC201 induced the decline of regulatory proteins of mtDNA transcription and translation (Figure 5C). Similarly to the above-discussed changes, short-term and long-term treatment of BT474 cells with single-dose ONC201 resulted in reversible and irreversible suppression of the expression of proteins involved in the regulation of transcription and translation factors of mitochondrial DNA (Figure 5C). Cells exposed to ONC201 for 24 h and longer resulted in an almost complete, fast (24 h), and persistent degradation of EF-Tu, mitochondrial translation elongation factor Tu, required for mtDNA translation [28,46], and 120 h washout of cells in drug-free media did not restore this protein. The effect of ONC201 treatment on the level of TFAM, mitochondrial transcription factor A, a protein determining mtDNA packaging and transcription [28,47,48], was gradual, and degradation increased with the initial exposure to the drug (Figure 5B, TFAM). The 120 h washout restored the expression of TFAM only in cells exposed to ONC201 for 24 h and 48 h (Figure 5C, TFAM). The pattern of expression of mtDNA transcription and translation proteins correlated with the expression of CLPP and its partner CLPX (Figure 5C). Mitochondrial caseinolytic peptidase, CLPP, is the only known up-to-date intracellular target of ONC201, which activates this protease even in the absence of CLPP partner CLPX [3,4,5]. Our observation indicates that degradation of mitochondrial nucleoids and mtDNA could be due to CLPPX-mediated degradation of structural protein TFAM, the suppression of mitochondrial respiratory chain activity leading to cell cycle arrest at the G_1_/S checkpoint (Figure 5 and Figure 6). Interestingly, the transient treatment of BT474 cells with single-dose ONC201, resulting in cell cycle arrest, inhibition of proliferation and persistent cellular stress, and inhibition of proliferation, was associated with suppression of staurosporine-induced apoptotic cell death, in line with our observation of the decline in pro-and anti-apoptotic proteins. The most interesting outcome of ONC201 treatment of cells is that this compound also induces transformation of BT474 breast cancer cells into stress- and/or therapy-induced cellular senescence-like phenotypes, resulting in modification of the sensitivity of these transformed cells toward NK-cell mediated killing. NK cells, which are activated by an array of stressor and senescence- associated determinants, play an instrumental role in immunosurveillance and senescent cell elimination [49]. Our findings point to the feasibility of a complementary therapeutic mechanism of ONC201 and NK cells via ONC201-induced upregulation of endogenous NK cell anti-tumor activity. This synergistic effect suggests a possible benefit of cancer treatment by ONC201 in combination with NK-based cellular immunotherapies [50].

In summary, ONC201, through activation of mitochondrial matrix CLPP and irreversible degradation of mitochondrial TFAM and destabilization of mtDNA, leads to the loss of mtDNA and nucleoids. This in turn leads to the loss of the vital subunits of the mitochondrial respiratory chain encoded by mtDNA, and impairment of oxidative phosphorylation. Inhibition of mitochondrial oxidative phosphorylation leading to energy deprivation of BT474 cells exposed to ONC201 induces cell cycle arrest, paralleled with suppression of cyclin E and Cdk2 expression, markers of the cell cycle arrest. The differences in the expression of Cyclin E and CDK2 induced by ONC201 are very prominent and need further investigation. ONC201 also induces transformation of BT474 breast cancer cells into stress- and/or therapy-induced cellular senescence-like phenotypes, resulting in modification of the sensitivity of these transformed cells toward NK-cell mediated killing, similarly to observations described by other investigators. These senescence-like states of BT474 cells induced by ONC201 treatment warrant further investigations into molecular mechanisms involved in the transformation of BT474 cells through ONC201-induced activation of mitochondrial ClpP.

## 4. Materials and Methods

### 4.1. Chemicals

ONC201 was obtained from SelleckChem (S7963, Houston, TX, USA). Other chemicals used were from Sigma-Aldrich (St. Louis, MO, USA or as indicated). The concentration of the vehicle (DMSO) used as a solvent for hydrophobic agents was kept under 0.5%.

### 4.2. Cell Culture

Human breast cancer cell line BT474 (obtained from Russian Cell Culture Collection, Institute of Cytology, Russian Academy of Sciences, St. Petersburg, Russia RCCC, Russia) was cultured in Dulbecco’s modified Eagle’s medium (D5648, Sigma-Aldrich, St. Lois, MO, USA), supplemented with 10% fetal bovine serum (F9665, Sigma-Aldrich, St. Lois, MO, USA), 2.2 g/L NaHCO_3_ (S5761, Sigma-Aldrich, St. Lois, MO, USA) and a 1% mixture of Antibiotic–Antimycotic (A5955, Sigma-Aldrich, St. Lois, MO, USA) in a cell culture incubator set at Air 95%/CO_2_ 5% and 37 °C.

### 4.3. Time-Course of the Typical Experiments of BT474 Cells Treated with a Single Dose of ONC201 (10 µM)

The effects of short-term (24 h) and long-term (72 h) single-dose treatment of BT474 cells with ONC201 and a subsequent 120 h washout of the drug was studied using the protocol shown in Supplementary Figure S1. Subsequent to short- and/or long-term treatments, cells were incubated for 120 h in drug-free media with every 24 h exchange of the incubation media (Supplementary Figure S1).

### 4.4. Evaluation of Dose-Dependent Effect of ONC201

Cells were plated in a 96-well plate (SPL Lifesciences, Korea) at a density of 10,000 cells/cm^2^ in triplicates and left to adhere overnight (14–16 h) in a cell culture incubator. The next-day incubation media was replaced with another one supplemented with different doses of ONC201 (0–50 µM), and cells were incubated in the presence of the drug for 24, 48, 72, and 96 h. After incubation, incubation media was collected from wells for further counting of floating cells. Cells in the wells were stained with 2 µg/mL Hoechst 33342 in Hank’s Balanced Salt Solution (HBSS) for 30 min in the cell culture incubator. After staining, cells were twice washed with fresh HBSS, and fluorescence of Hoechst 33342 was measured on fluorescence plate reader Infinite F Plex (Tecan, Grodig, Austria) in the following mode: excitation 340, emission 460; multiple 2 × 2 bottom reading. Collected incubation media with floating cells was spun at 900× *g* for 5 min; the cell pellet was resuspended in 50 µL of HBSS, and the number of cells was calculated using a hemocytometer. All floating cells were assumed to be dead, and the number of live cells was calculated using a calibration graph for Hoechst 33342 fluorescence.

### 4.5. Mitochondrial Respiratory Characteristics in Digitonin-Permeabilized BT474 Cells

Adhered and flattened BT474 cells treated with ONC201 were trypsinized and washed once with PBS as described [4]. Pelleted cells (300× *g*, 5 min, RT) were suspended in a respiration buffer (mM): 110 KCl, 5 NaCl, 5 KH_2_PO_4_, 10 HEPES, pH 7.4, supplemented with 5 glutamate + 5 pyruvate (Complex I) or 10 succinate + 0.001 Rotenone (Complex II). Aliquots of suspension, containing 4 × 10^6^ cells/mL, were transferred into the measuring chamber of multichannel recorder FluoFlux Æ1 (Econix-Expert Inc., Moscow, Russian Federation, webpage: ionomer.ru) and an oxygen sensor designed as described earlier [51]. Following permeabilization of cells with 0.005% digitonin, an aliquot of ADP (200 µM) was added to initiate oxidative phosphorylation (State 3 respiration). After reaching the steady-state level, State 3 respirating cells were treated with 10 µM Carboxyatractyloside, an inhibitor of ANT, the adenine-nucleotide transporter, to inhibit oxidative phosphorylation, and, subsequently, the maximal rate of cellular respiration was induced with 25 µM of 2,4-DNP.

### 4.6. Caspase 3/7 Activity Assay

Cells were plated at 30,000 cells/cm^2^ in a 96-well plate and left to adhere overnight in a cell culture incubator, and the next day were treated for indicated times with 10 μM ONC201 or 0.5 µM staurosporine. Following incubation for the indicated time and removal of the incubation media, 100 µL of lysis buffer, containing 50 mM HEPES, 5 mM CHAPS, and 5 mM DTT, pH 7.4, were added to cells. Caspase activity was measured as described earlier [4].

### 4.7. Cell Cycle Analysis by Flow Cytometry

BT474 cells were plated on 90-mm Petri dishes (10,000 cells/cm^2^) and treated with ONC201 as indicated in Supplementary Figure S1. After treatment, cells were harvested by trypsinization, washed once with PBS (300× *g*, 5 min, RT), and transferred into ice-cold 70% ethanol and stored at −20 °C for at least 24 h. After fixation, cells were twice washed from ethanol in PBS (900× *g*, 5 min, RT). After that, cells were suspended and incubated in PBS, containing 10 µg/mL of Propidium Iodide and 100 µg/mL RNAse (Sigma-Aldrich) for 15 min at 37 °C. The distributions of cells between G_0_/G_1_, S, and G_2_/M phases of the cell cycle were evaluated using BD Accuri C6 (BD Bioscience, San Jose, CA, USA), and obtained data were analyzed using ModFit LT 4.1 software.

### 4.8. Confocal Microscopy and Image Analysis of Mitochondrial Nucleoids and Mitochondrial Size

For confocal microscopy experiments, BT474 cells were plated on 35-mm Petri dishes (15,000 cells/cm^2^) and treated with appropriate drugs as indicated in the figure legends and/or in the text. Cells rinsed three times with 2 mL of HBSS buffer were incubated in 2 mL of HBSS buffer, supplemented with 2 µg/mL Hoechst 33342 (H3570, Invitrogen, Waltham, MA, USA), SYBR Green I at dilution of 1:200,000 (S7563, Invitrogen, USA), and 150 nM MitoTracker Deep Red 633 (M-22426, Molecular Probes, Eugenius, OR, USA) at 37 °C for 30 min in a CO_2_-free thermostat. Following staining, the cells were rinsed 3 times with dye-free HBSS, and fluorescent images of cells were obtained using fluorescent scanning confocal microscope Leica TCS SP-5 DM6000 CS (Leica Microsystems, Wetzlar, Germany) at a sequential scanning mode using HCX PL APO lambda blue 63×, NA = 1.4 (Leica Microsystems, Wetzlar, Germany). Excitation and emission were set for Hoechst 33342 405 nm/460 nm, SYBR Green I 488 nm/540 nm, and MitoTracker Deep Red 633 633 nm/710 nm. Quantification of the number of mitochondrial nucleoids and mitochondrial size was performed using the fluorescence confocal images of BT474 cells labeled for mtDNA (stained with SYBR Green-1) and mitochondrial mass (stained with MitoTracker Deep Red). Images were analyzed using the Fiji software algorithm [52] and macros described in [12].

### 4.9. Measurement of mtDNA by qPCR

Cells subjected to appropriate treatment were trypsinized using a standard trypsin/EDTA solution, rinsed once with PBS, and suspended at a concentration of 1.5 × 10^6^ cells/mL of ice cold hypotonic RSB buffer: 10 mM NaCl, 1.5 mM MgCl_2_, 10 Tris-HCl, pH 7.5 [53]. After allowing cells to swell for 15 min on ice, 200 µL of cell suspension (containing 300,000 cells) were used for extraction and analysis of the mtDNA/nDNA ratio. Briefly, total DNA was extracted using the K-Sorb kit (Sintol, Moscow, Russia), and quantitative analysis of mtDNA was carried out by real-time PCR with TaqMan oligonucleotides on a Prism 7500 thermal cycler (Applied Biosystems, Foster City, CA, USA) as described [54]. The relative quantity of mtDNA/nDNA was determined as a ratio between the number of copies of the mitochondrial ND1 gene and that of the β-actin gene of nDNA in the same test tube. PCR tests were carried out in triplicate for each DNA sample using the following primers: for ND1:forward—5′-CCC CTA AAA CCC GCC ACA TC-3′; reverse—5′-GTA GAA GAG CGA TGG TGA GAG C-3′, and a probe—R6G-AC CCT CTA CAT CAC CGC CCC GAC C-BHQ1. For amplification of the β-actin gene, the following primers were used: forward—5′-TCA CCC ACA CTG TGC CCA TCT ACG A-3′; reverse—5′-TCG GTG AGG ATC TTC ATG AGG TA-3′, and a probe—FAM-AT GCC CTC CCC CAT GCC ATC C-RTQ1. The protocol used was as follows: 5 min at 95 °C followed by 40 cycles (95 °C for 30 s, annealing and elongation at 60 °C for 1 min). Obtained data were analyzed and presented as a percentage of data compared to non-treated BT474 cells using methods of relative gene expression as described [54,55].

### 4.10. Immunoblotting

For Western blotting, BT474 cells were plated on 90-mm Petri dishes (2 × 10^6^ cells per plate) and treated with ONC201 as described above. Following treatment, cells were trypsinized using a standard trypsin/EDTA solution, then rinsed 3 times with 2 mL of ice-cold PBS, and lysed using RIPA lysis buffer (Santa Cruz, CA, USA) supplemented with 1 mM Na(VO_3_)_4_, 2 mM PMSF, and a complete protease inhibitor cocktail (Santa Cruz, CA, USA). After sonication and clarification by centrifugation at 14,000× *g*, the protein concentration in supernatants was measured with the Bradford method; lysates were mixed with 4X Laemmle’s loading buffer, boiled for 5 min, and 30 µg of each protein lysate sample was subjected to polyacrylamide gel electrophoresis (BioRad, Hercules, CA, USA) followed by electro blotting to the nitrocellulose membrane. Membranes were blocked with 5% milk/TBST at room temperature for 1 h and incubated overnight at 4 °C using appropriate primary antibodies in TBST/5% BSA + 0.02% NaN_3_. The used antibodies were: CHOP (Cell Signaling, Danvers, MA, USA, 1:500), ATF4 (Cell Signaling, Danvers, MA, USA, 1:1000), p-AMPK (Cell Signaling, Danvers, MA, USA, 1:1000); or in TBST/5% milk: GDF-15 (Santa Cruz, CA, USA, 1:1000), TFAM (Santa Cruz, CA, USA, 1:1000), TUFM (Invitrogen, 1:1000), ClpP (Cell Signaling, 1:1000), ClpX (Invitrogen, 1:1000), Cyclin E (Santa Cruz, CA, USA, 1:1000), Cdk2 (Santa Cruz, CA, USA, 1:1000), Lamin B1 (Abcam, Cambridge, UK, 1:1000), HMGB1 (Abcam, Cambridge, UK, 1:1000), (GAPDH, Santa Cruz, CA, USA, 1:1000). Following overnight incubation with primary antibodies at 4 °C, membranes were washed 3 × 15 min in TBST, and placed for 1 h in their respective 2° antibodies (1:10,000 dilution in 5% milk/TBST). HRP-labeled membranes were washed for 3 × 15 min in TBST and embedded in 2 mL of ECL reagent, and images of immunoreactive bands were obtained using the chemiluminescent method and Clarity Western ECL substrate (Bio-Rad, Hercules, CA, USA). The Chemidoc Touch Imaging System and Image Lab software (Bio-Rad) were used for the processing/quantification of obtained images.

### 4.11. NK Cytotoxicity Assay

ONC201-treated cells were exposed to isolated human NK cells purified from whole blood samples obtained from healthy donors as described [56]. Briefly, peripheral blood mononuclear cells (PBMCs) were purified from whole blood by the Ficoll-Hypaque density gradient centrifugation using the BD Vacutainer CPT Cell Preparation Tubes (Becton, Dickinson and Company, Franklin Lakes, NJ, USA). NK cells were magnetically separated from PBMCs with the NK cell isolation kit (Miltenyi Biotec, Bergisch Gladbach, Germany) according to the manufacturer’s instructions. Purified NK cells were expanded in NK MACS medium (Miltenyi Biotec, Bergisch Gladbach, Germany) supplemented with 5% autologous plasma and IL2 (500 IU/mL) for 12 days in a humidified air atmosphere containing 5% CO_2_ at 37 °C. NK cells were added to target cells, and the target cell viability was determined by the neutral-red uptake method with modifications [57]. In brief, the plates are incubated for 30 min with PBS containing Neutral Red (80 μg/mL); the accumulated dye was extracted from the viable cells using sodium dodecyl sulfate solution, and the absorbance was measured by a spectrophotometer at 540 nm. The cell viability value was estimated as a percentage of viable cells after treatment with NK cells (untreated cells were taken as 100%).

### 4.12. TRAIL Cytotoxicity Assay

Human recombinant TRAIL (TNF-related apoptosis inducing ligand) and DR5-B (TRAIL mutant, selective against DR5 receptor [58]) were kindly provided by M. E. Gasparyan from Shemyakin-Ovchinnikov IBCh RAS. BT474 cells were plated in a 96-well cell culture plate and treated with ONC201 as described above. After ONC201 treatment/washout, the incubation medium was replaced with another one (control), supplemented with 1000 ng/mL TRAIL and/or 10 ng/mL DR5b, and cells were incubated with TRAIL or DR5b for 24 h. After that, 10 µL of resazurin solution in PBS (1 mg/mL) were added to wells, and the rate of resazurin reduction was measured fluorometrically using TECAN F PLEX (excitation 532 nm, emission 595 nm). The viability of cells (for each ONC201 treatment condition) was estimated as a ratio of resazurin reduction rates in the TRAIL- or DR5b-treated cells to the untreated (control) cells.

### 4.13. Statistical Analysis

The results of the present study presented as the mean ± SD, and all experiments were carried out at least 3 times. Comparison of the results between the control (in the absence of agents) and experimental groups (in the presence of agents) was performed using the paired Student’s and/or ANOVA with the post-hoc Bonferroni test, and *p* < 0.05 was considered as statistically significant.

## Data Availability

Data are available within the article and Supplementary Materials.

## Supplementary Materials

The supporting information can be downloaded at: https://www.mdpi.com/article/10.3390/ijms232415551/s1.
