# Supplementary material for: ONC201-Induced Mitochondrial Dysfunction, Senescence-like Phenotype, and Sensitization of Cultured BT474 Human Breast Cancer Cells to TRAIL
Source: Int J Mol Sci. 2022 Dec 8;23(24):15551. doi: 10.3390/ijms232415551 (PMC9779726; doi:10.3390/ijms232415551)
Supplement: Supplementary file 1 [file ijms-23-15551-s001.zip › ijms-2033286-supplementary.pdf]

Supplementary Materials:

ONC201-Induced Mitochondrial Dysfunction, Senescence-like Phenotype, and Sensitization of Cultured BT474 Human Breast Cancer Cells to TRAIL

Artem Mishukov [1,2], Irina Odinokova [1], Ekaterina Mndlyan [1], Margarita Kobyakova [1], Serazhutdin Abdullaev [1], Vitaly Zhalimov [3], Xenia Glukhova [1], Vasiliy Galat [4], Yekaterina Galat [4], Anatoly Senotov [1], Roman Fadeev [1], Artem Artykov [5], Marine E. Gasparian [5], Marina Solovieva [1], Igor Beletsky [1,*] and Ekhson Holmuhamedov [1,*]

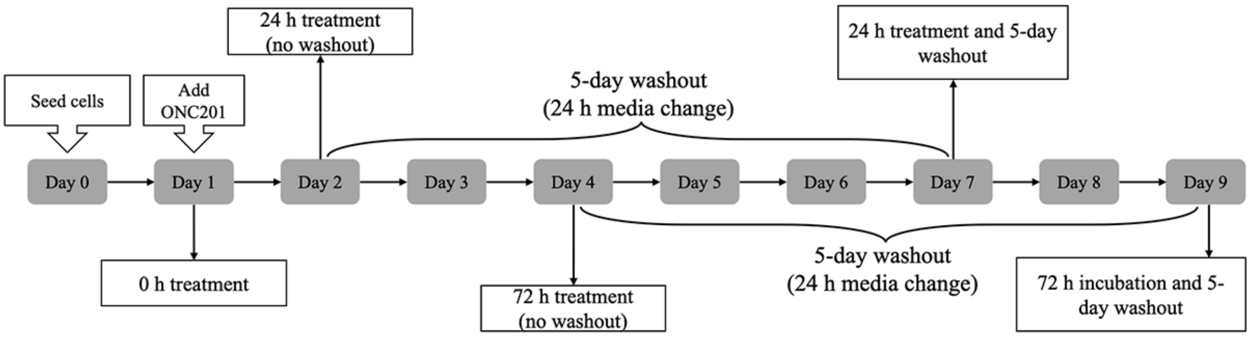

Figure S1. The diagram demonstrating treatment of BT474 cells with ONC201 for evaluation of long-lasting consequences.

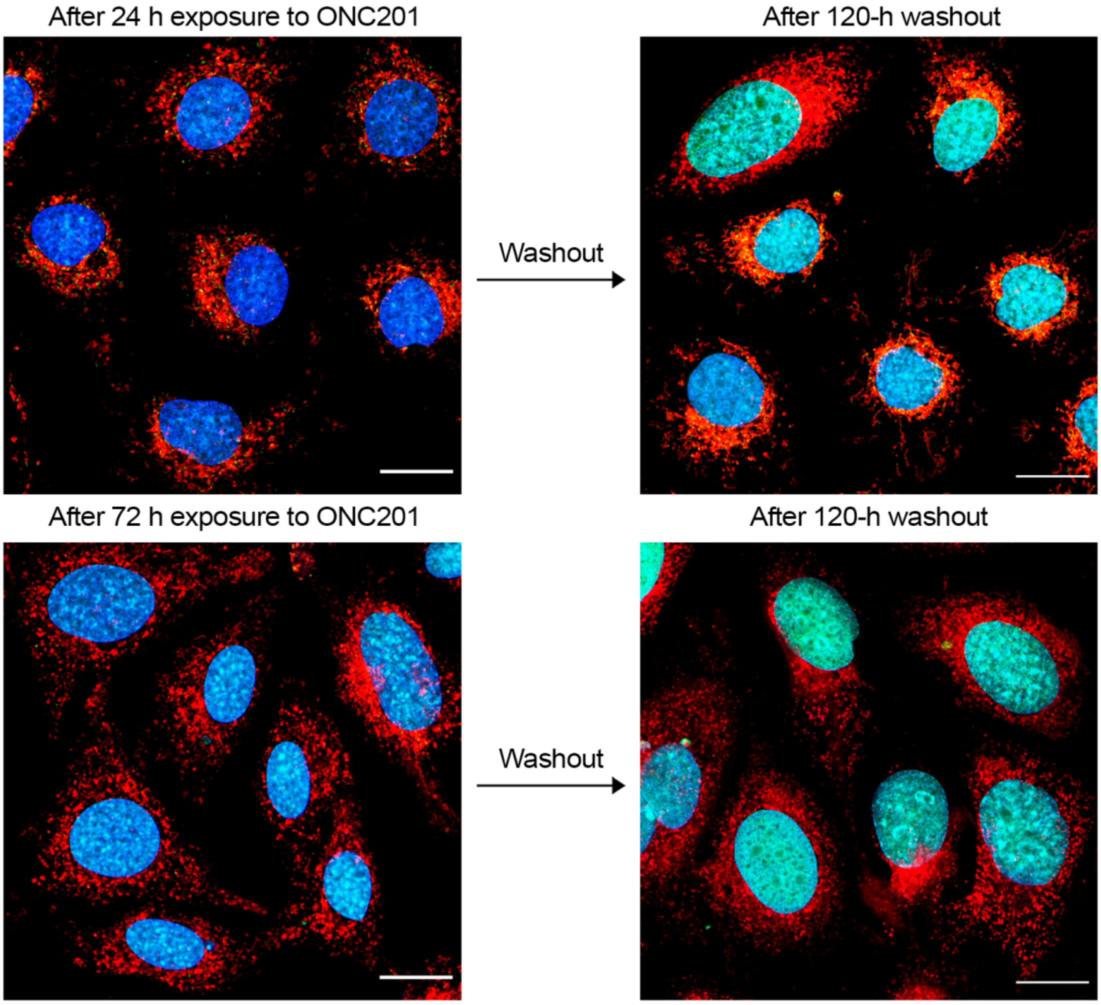

Figure S2. Confocal images of BT474 cells after 24 h, 72 h exposure to ONC201 and subsequent 120-h washout. Cells were treated with ONC201, stained with dyes (Hoechst, SYBR Green I, MitoTracker Deep Red) and images were collected as described in the text.

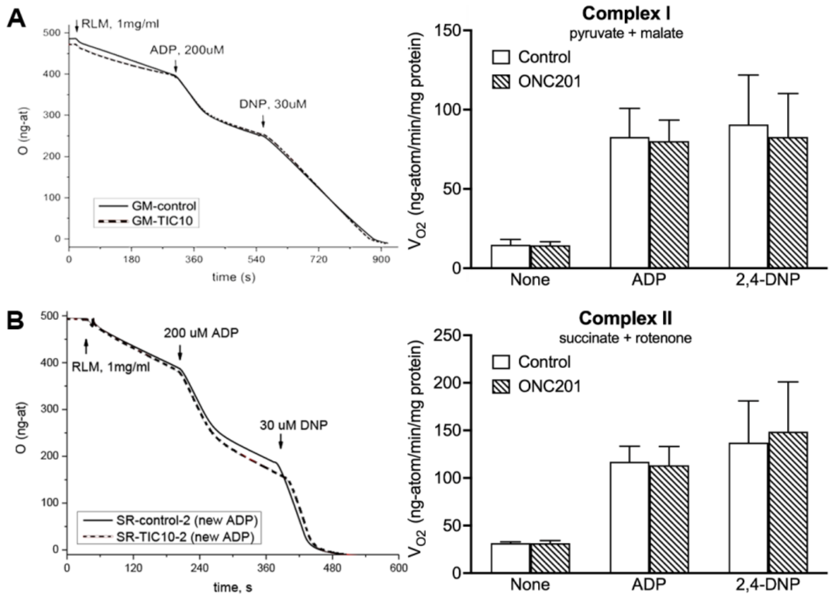

Figure S3. The effect of ONC201 on the major respiratory characteristics of isolated mitochondria. **A,** Complex I supported respiration and basal respiratory characteristics (None, ATP- and 2,4-DNP stimulated) of isolated mitochondria. **B,** Complex II supported respiration and basal respiratory characteristics (None, ATP- and 2,4-DNP stimulated) of isolated mitochondria. Incubation media contained: 110 mM KCl, 5 mM NaCl, 2 mM KH_2PO_4, 10 mM Tris (pH 7.4), and 5mM glutamate and 5 mM malate (GM, **A**) or 10 mM succinate and 2.5 uM rotenone (SR, **B**), and 1 mg mitochondrial protein/ml . Presented data are from at least 4 independent measurements.
